# Development and Delivery of a Plastic and Reconstructive Surgery Case-Based Learning Curriculum for Pre-Clerkship Medical Students

**DOI:** 10.1177/22925503261470691

**Published:** 2026-07-30

**Authors:** Dave Gwun, Victoria Tucci, Alexandra D'Souza, Thomas Milazzo, Sophocles Voineskos, David Wallace, Kyle R. Wanzel, Shaishav Datta

**Affiliations:** 1 Temerty Faculty of Medicine, 12366 University of Toronto, Toronto, ON, Canada; 2 Department of Surgery, Division of Plastic, Reconstructive & Aesthetic Surgery, 12366 University of Toronto, Toronto, ON, Canada; 3 Li Ka Shing Knowledge Institute, Unity Health Toronto, Toronto, ON, Canada

**Keywords:** Career choice, case-based learning (closest MeSH: problem-based learning), education, medical, undergraduate, plastic and reconstructive surgery (closest MeSH: surgery, plastic), Choix de carrière, apprentissage par cas, enseignement médical, premier cycle, chirurgie plastique et reconstructive, apprentissage par problèmes, chirurgie plastique

## Abstract

**Introduction:**

Canadian medical students receive limited exposure to Plastic and Reconstructive Surgery (PRS) during pre-clerkship. To address this gap, a longitudinal PRS case-based learning (PRS-CBL) curriculum was developed to parallel the pre-clerkship curriculum, with an aim to increase early exposure to PRS concepts.

**Methods:**

Guided by Medical Council of Canada objectives, PRS faculty and residents co-designed a 5-part case-based learning curriculum: Introduction to PRS, Skin and Soft Tissue Infections, Benign and Malignant Skin Lesions, Hand and Wrist Trauma, and Burns. Each session combined a didactic presentation, clinical case with facilitated discussion, and hands-on workshop. Eligible participants were first- and second-year medical students. Presession and postsession tests were compared using the Mann-Whitney *U* test. Written feedback was analyzed using thematic analysis.

**Results:**

From 2023 to 2025, 10 PRS-CBL sessions were delivered, with a mean of 45 (SD 28.8) registrants per session. Of 453 total registrants, 201 were unique participants, with 41% reporting no prior interest in PRS. Significant improvements in presession versus postsession test scores were observed at each session (*p* < .05), except for session 4. Over 83% of respondents strongly agreed that the learning objectives were met, content was appropriate, and sessions were highly effective. Qualitative themes highlighted the value of hands-on, multimodal teaching and opportunities to interact with PRS faculty and residents.

**Conclusion:**

The PRS-CBL curriculum filled a void in the current undergraduate medical curriculum, improved PRS knowledge, received favorable evaluations, and included students without prior interest in PRS. This adaptable model may support early career exploration in PRS and other underrepresented specialties.

## Résumé

**Introduction:**

Les étudiants en médecine canadiens n’ont qu’une exposition limitée à la chirurgie plastique et reconstructive (CPR) pendant la phase préclinique. Afin de combler cette lacune, un programme d’enseignement longitudinal de la CPR basé sur des cas cliniques (CPR-CBL) a été élaboré en parallèle du programme préclinique, dans le but d’accroître l’exposition précoce aux concepts de la CPR.

**Méthodes:**

En s’appuyant sur les objectifs du Conseil médical du Canada, les enseignants et les résidents en chirurgie plastique et reconstructrice ont conçu conjointement un programme d’apprentissage par cas en cinq parties : Introduction à la chirurgie plastique et reconstructrice, Infections de la peau et des tissus mous, Lésions cutanées bénignes et malignes, Traumatismes de la main et du poignet, et Brûlures. Chaque session combinait un exposé théorique, une étude de cas clinique suivie d’une discussion animée, et un atelier pratique. Les participants éligibles étaient des étudiants en médecine de première et deuxième année. Les tests réalisés avant et après les sessions ont été comparés à l’aide du test U de Mann-Whitney. Les commentaires écrits ont été analysés par analyse thématique.

**Résultats:**

Entre 2023 et 2025, 10 sessions PRS-CBL ont été dispensées, avec une moyenne de 45 inscrits (écart-type : 28,8) par session. Sur un total de 453 inscrits, 201 étaient des participants uniques, dont 41 % ont déclaré n’avoir manifesté aucun intérêt préalable pour la CPR. Des améliorations significatives des scores aux tests pré et post-session ont été observées à chaque session (p < 0,05), à l’exception de la session 4. Plus de 83 % des répondants ont affirmé qu’ils étaient tout à fait d’accord pour dire que les objectifs d’apprentissage avaient été atteints, que le contenu était approprié et que les sessions étaient très efficaces. Les thèmes qualitatifs ont mis en évidence la valeur d’un enseignement pratique et multimodal, ainsi que les opportunités d’interaction avec le corps enseignant et les internes en CPR.

**Conclusion:**

Le programme CRR-CBL a comblé une lacune dans le programme actuel de médecine de premier cycle, a amélioré les connaissances en chirurgie plastique et reconstructive, a reçu des évaluations favorables et a inclus des étudiants qui ne s’intéressaient pas auparavant à cette spécialité. Ce modèle adaptable pourrait favoriser l’exploration précoce des débouchés professionnels en chirurgie plastique et reconstructive ainsi que dans d’autres spécialités sous-représentées.

## Introduction

Medical students receive limited formal exposure to Plastic and Reconstructive Surgery (PRS) during undergraduate medical training.^1–3^ A 2015 survey conducted at our institution reported that nearly 85% of fourth-year medical students had not received formal PRS instruction, and 90% felt unprepared to manage common conditions treated by plastic surgeons.^4^ Similar trends have been noted in the literature, where PRS remains one of the least represented specialties in medical curricula, with many students receiving fewer than 2 hours of structured teaching throughout their medical training.^3,5,6^ This limited exposure may affect students’ understanding of PRS and early clinical decision-making, as the scope of the specialty is often poorly recognized by trainees and primary care physicians, potentially contributing to delays in consultation or referral.^1,5,7–11^ Early exposure to PRS has also been suggested to support informed specialty decision-making and the development of clinically relevant skills transferable across disciplines.^4,12–15^

Several strategies have been implemented to increase undergraduate exposure to PRS, including short stand-alone lectures, longitudinal seminar series, clinical observerships, and student-led interest groups.^16–21^ During the coronavirus disease 2019 (COVID-19) pandemic, digital platforms further expanded access through livestreamed surgeries, online modules, and virtual electives.^22–28^ Despite demonstrated interest and educational value, PRS education remains largely voluntary or extracurricular and is not systematically integrated into undergraduate medical curricula. A previous Letter to the Editor by Datta et al^23^ described the development of a case-based learning (CBL) approach to PRS content delivery at our institution in response to limited undergraduate PRS exposure and the educational disruptions associated with the COVID-19 pandemic. The CBL approach was selected because it is a well-established educational model that promotes critical reasoning, communication, and application of knowledge through small-group clinical case analysis.^29–32^ As CBL is already well integrated into pre-clerkship education at our institution, it represented a natural extension of the existing curricular structure.

To address the need for more structured and accessible early PRS exposure in undergraduate medical education, this pilot CBL approach was expanded and formalized into a longitudinal PRS-CBL program. The curriculum was designed to provide early, multimodal, and structured exposure to PRS for first- and second-year medical students in a format that complemented the existing pre-clerkship curriculum. This study aimed to describe the development and delivery of this program, evaluate its effectiveness as an educational initiative with 2 academic years of evaluative data, and provide a critical reflection on our experiences throughout implementation.

## Methods

This study received approval from University of Toronto's Institutional Research Ethics Board (REB #46300). The Guideline for Reporting Evidence-based practice Educational interventions and Teaching (GREET) checklist guided study design and rigor (Supplemental Material S1).^33^

### Pilot PRS-CBL Series

The pilot series by Datta et al,^23^ consisting of 9 sessions, was conducted from February to June 2021. Each session included 30 min of didactic teaching and 30 min of assigned group questions, followed by a tutor-led review and peer-to-peer teaching. Attendance ranged from 7 to 14 first- and second-year medical students. After each session, students provided feedback for quality improvement, which was incorporated into the present curriculum.^23^

### Curriculum Development

Following the pilot study, the PRS-CBL curriculum was refined through iterative discussions among PRS faculty and residents involved in curriculum development and delivery to create a more structured, standardized, and longitudinal program. Lecture slides, clinical cases, and workshop components underwent multiple rounds of review and revision to improve consistency, relevance, and appropriateness for pre-clerkship learners.

During the 2021 pilot, sessions were evaluated through postsession surveys that included Likert-scale items assessing session quality, content, and delivery, as well as open-ended prompts for qualitative feedback. These responses were reviewed by the study team to identify recurring strengths and areas for improvement.

Feedback from the pilot directly informed several modifications to the final curriculum, including reducing the number of sessions from 9 to 5 to prioritize high-yield topics, incorporating hands-on procedural workshops into each session to enhance interactivity, and aligning session timing more closely with the pre-clerkship academic schedule. The 5 sessions each cover a high-yield PRS topic: (1) Introduction to PRS (ie scope of practice, addressing common misconceptions, and foundational topics including the reconstructive ladder and the stages of wound healing), (2) Skin and Soft Tissue Infections, (3) Benign and Malignant Skin Lesions, (4) Hand and Wrist Trauma, and (5) Burns. Topics were selected based on survey responses from PRS faculty who identified the most appropriate topics for pre-clerkship medical students. Topic selection was further guided by the learning objectives and competencies assessed on the Medical Council of Canada Qualifying Examination (MCCQE) (Table 1).^34^

**Table 1.**
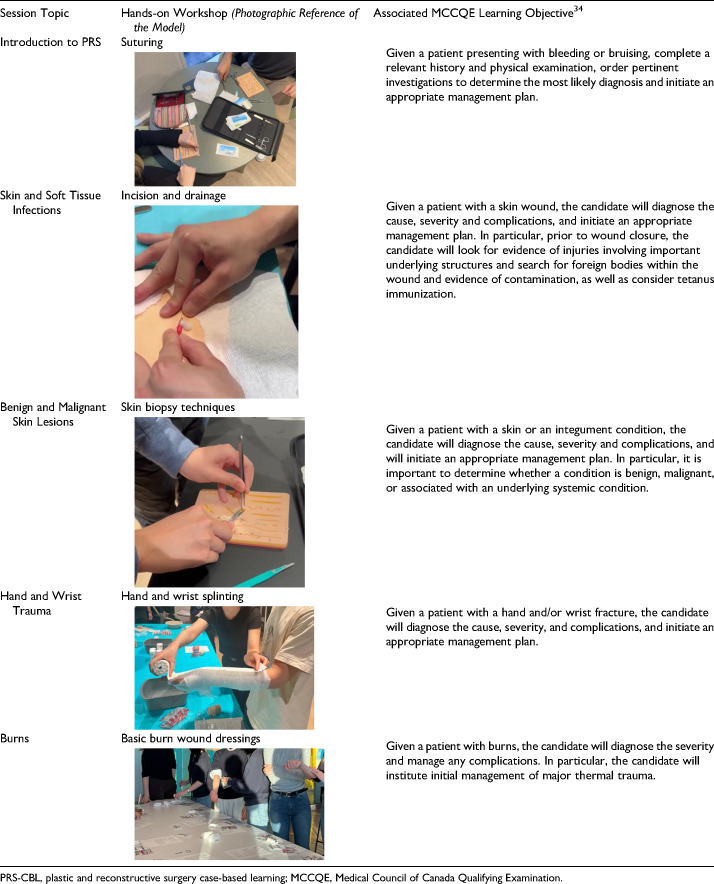
PRS-CBL Session Topics with Associated Hands-on Workshops Mapped to the Corresponding MCCQE Learning Objective.

All course materials, including (1) predeveloped and standardized didactic lecture slides, (2) clinical cases and associated questions and answers, (3) hands-on procedural workshops, (4) presession and postsession knowledge assessments, and (5) postsession feedback surveys were developed by senior medical students in collaboration with PRS residents and faculty at our institution to ensure educational quality and appropriateness for learner level.

Standardized lecture slides for didactic teaching were developed to emphasize core principles, including anatomy, focused history-taking, physical examination techniques, diagnostic features, investigations, and management principles for common PRS presentations. Clinical vignettes with associated question sets were created to consolidate lecture material through collaborative, small-group problem-solving in a CBL format to emphasize active engagement while providing structured, targeted objectives.^32^ Faculty or resident facilitators reviewed all cases and answers with student groups. Interactive procedural workshops were designed to introduce generalist-level technical skills, including (1) suturing, (2) incision and drainage, (3) skin biopsy techniques, (4) hand and wrist splinting, and (5) basic burn wound dressings. Standardized handouts summarizing procedural steps were distributed for independent practice, and all workshop materials were provided free of charge.

### Curriculum Delivery

**Figure 1.**
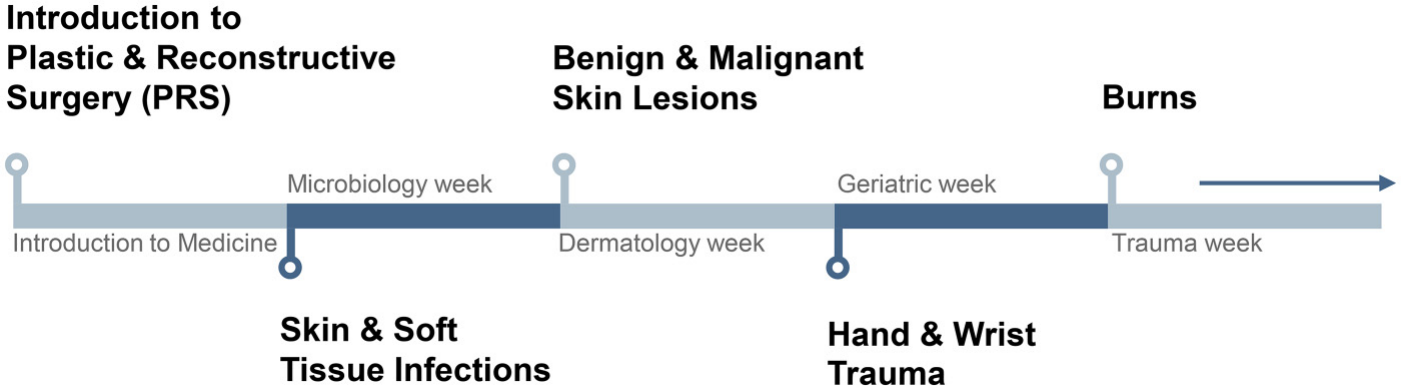
Timeline of plastic and reconstructive surgery case-based learning (PRS-CBL) sessions overlaid with first and second-year pre-clerkship curriculum topics.

Sessions were delivered in-person throughout the 2023 to 2024 and 2024 to 2025 academic years (from September to May) and were intentionally scheduled to parallel and complement the existing pre-clerkship curriculum at our institution (Figure 1). The in-person delivery model was selected to foster community, mentorship, and direct engagement, while also countering postpandemic “Zoom fatigue.”^35^ These sessions were typically scheduled during evening hours (eg 18:00-20:00).

PRS residents and faculty members who were previously involved in curriculum development or had expressed interest in facilitation were invited to lead the sessions. Overall program functioning was overseen by 4 faculty collaborators. Each session typically included 1 staff PRS faculty facilitator, 3 resident facilitators (2 junior and 1 senior resident), and 2 senior medical students. Facilitators were provided with standardized, pre-developed session materials in advance, including the didactic slides and case materials, to support consistency in delivery. Each session was approximately 2 h in duration and followed a standardized structure that began with a 30- to 45-min lecture. Students were then divided into 2 groups and rotated through the case discussion and procedural workshop. During the case discussions, students worked collaboratively in groups of 5 to 10 and were encouraged to consult external resources, such as UpToDate^36^ and Orthobullets.^37^ Across sessions, the approximate learner-to-instructor ratio was 4:1. All case questions were subsequently reviewed with a resident or faculty facilitator, and workshops were supervised by faculty, residents, and senior medical student facilitators. Following the formal components of the sessions, many students stayed to ask additional questions and network with peers, residents, and faculty.

Towards the end of each session, students were given dedicated time to complete the postsession survey. To incentivize participation, students who attended at least 4 of the 5 sessions and completed the postsession surveys received a certificate of completion signed by PRS faculty members at our institution, including the Program Director of the Division of Plastic, Reconstructive and Aesthetic Surgery, which outlined their involvement in the initiative.

### Study Participants and Recruitment

All first- and second-year medical students and preclinical MD-PhD students at the University of Toronto were eligible to participate, representing approximately 600 students in any given academic year. Exclusion criteria included nonmedical students, third- and fourth-year medical students, and students from other institutions.

Students were recruited through institutional automated email announcements, follow-up emails to previous participants, and social media platforms. Posts advertising the initiative were shared in several student Facebook groups using the authors’ accounts, as well as on our initiative's Instagram account (@prs.cbl). Registration for each session was collected on a rolling basis through an online form, and prior attendance was not required for subsequent participation. All participants provided informed consent, and all data were de-identified after collection.

### Presession and Postsession Surveys and Knowledge Tests

Presession surveys included a registration form capturing students’ level of training and specialty of interest, as well as a presession knowledge quiz relevant to the session topic. The presession survey was a requirement for registration and attendance (Supplemental Material S2).

Postsession surveys included a 5-point Likert-scale evaluation of the session, an open-ended feedback question, and a postsession knowledge quiz (Supplemental Material S3). Postsession survey responses were anonymous to ensure the accuracy and impartiality of the results.

Procedural skills were not formally assessed using objective performance-based measures; therefore, references to “skill development” in the qualitative analysis reflect learners’ perceptions reported in open-ended feedback rather than direct assessment of procedural competence.

Presession and postsession knowledge assessments consisted of brief, session-specific formative quizzes developed by the curriculum team to reflect each session's core learning objectives. Quizzes generally included 3 questions per session, most commonly in multiple-choice format, with question formats tailored to content (eg image-based items, multiselect items, or short-answer response where appropriate) (Supplemental Material S3). The assessments were intended to sample foundational knowledge relevant to each topic rather than provide a comprehensive measure of competence. Identical presession and postsession questions were used to assess short-term changes in topic-specific knowledge.

### Statistical Analysis

Statistical analyses were performed using R (version 4.5.1).^38^ Continuous variables are reported as means (SD), and categorical variables as frequencies and percentages. Normality was assessed visually and using the Shapiro–Wilk test, which was rejected for all presession and postsession score distributions (all *p* < .001). Paired analysis (ie Wilcoxon signed-rank) could not be conducted for presession and postsession knowledge comparisons because postsession data were completely anonymous to protect students’ identities. Therefore, comparisons were performed using the Mann–Whitney *U* (ie Wilcoxon rank-sum) test. However, treating presession and postsession responses as independent may reduce statistical power.

Open-ended feedback responses were analyzed using thematic analysis, a widely used qualitative methodology that enables an in-depth understanding of participants’ perspectives.^39^ In accordance with Braun and Clarke, 2 authors reviewed all open-ended feedback responses.^40^ To minimize subjective bias and improve validity, each author independently coded responses, met to compare codes and discuss perspectives. Both authors independently coded responses by their valence (positive, unfavorable, mixed) and further subcategorized responses by references to specific features of the sessions. Related codes were then grouped into broader themes. All authors reviewed the themes to ensure internal coherence. Disagreements in interpretation were resolved by consensus among the authors.

## Results

### Demographics and Registration

Across all 10 sessions, 453 registration forms were submitted, representing 201 unique participants. In the 2023 to 2024 academic year, 104 registration forms were received from 64 unique participants, of whom 7 (11%) registered for 4 or more sessions. In 2024 to 2025, the number of registration forms increased to 349, accounting for 137 unique participants, with 38 students (28%) registering for 4 or more sessions.

**Figure 2.**
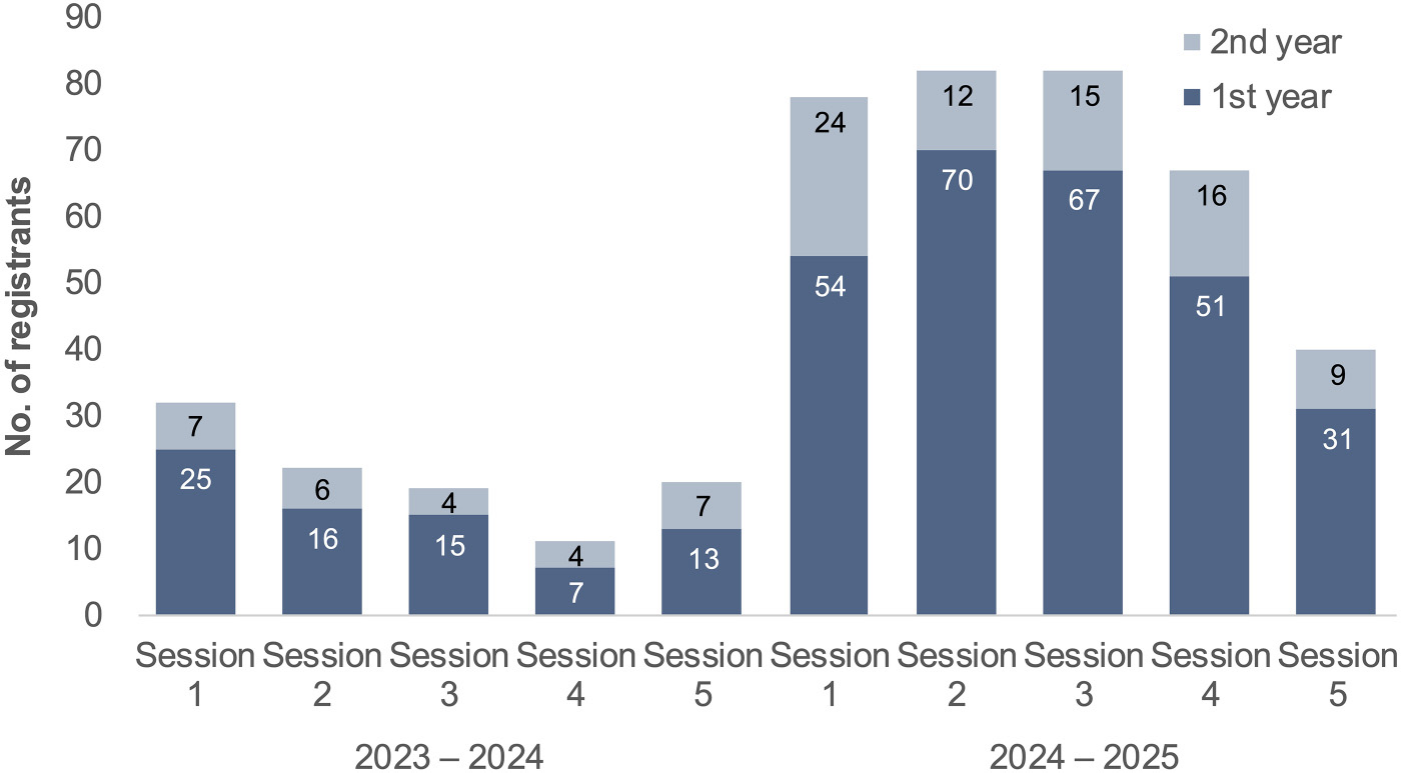
Number of registrants for sessions between September 2023 and May 2025.

On average, 45 students (SD 28.8) registered for each session, and the mean number of registrants per session increased from 21 (SD 7.5) in the first year of implementation to 70 (SD 17.8) in the second year. The majority of registrants were first-year medical students (77%). An overview of registrants per session is provided in Figure 2.

Participants reported 34 unique specialties or subspecialties of interest; 59% reported prior interest in PRS, followed by orthopedic surgery (12%), general surgery (10%), otorhinolaryngology (10%), and internal medicine (9%). Percentages reflect the proportion of participants selecting each specialty; totals may exceed 100%.

### Presession and Postsession Knowledge Tests

**Figure 3.**
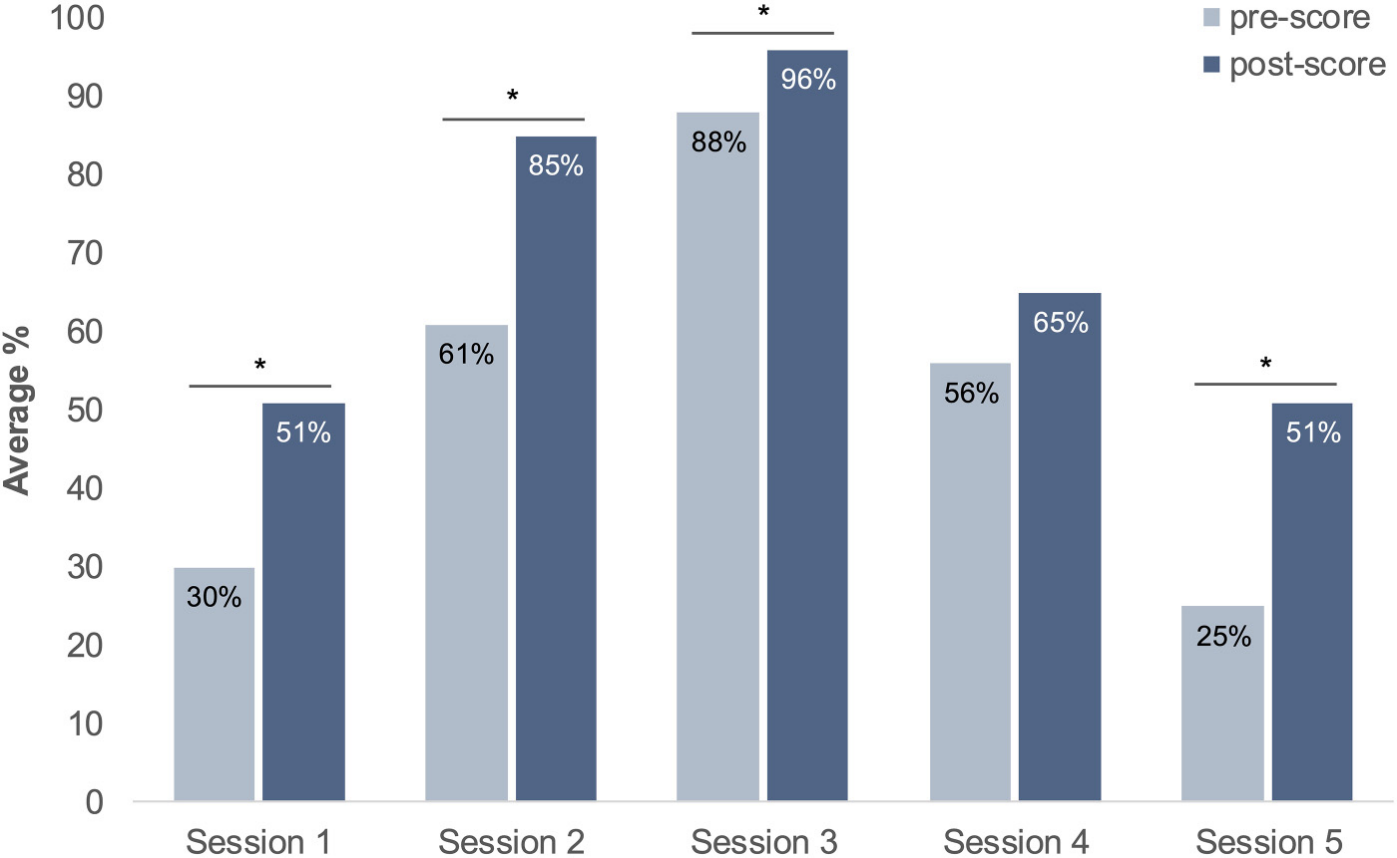
Presession and postsession knowledge mean test scores. Asterisk (*) denotes statistically significant difference (Mann-Whitney *U* test, *p* < .05).

Significant improvements in test scores were observed at each session (*p* < .05), except for session 4. Presession and postsession knowledge mean test scores are reported in Table 2 and Figure 3.

**Table 2.** Mean Presession and Postsession Knowledge Test Scores by Session.

Session	Presession Mean Score, % (SD)	Postsession Mean Score, % (SD)	*p*-Value*
Session 1	29.7 (27.2)	50.8 (25.4)	<.001
Session 2	60.6 (32.8)	85.4 (19.3)	<.001
Session 3	87.8 (21.5)	95.9 (12.7)	.003
Session 4	55.6 (29.7)	64.5 (27.6)	.094
Session 5	25.0 (23.5)	51.3 (21.4)	<.001

*p-values calculated using the Mann–Whitney U test.

### Session Evaluation

**Figure 4.**
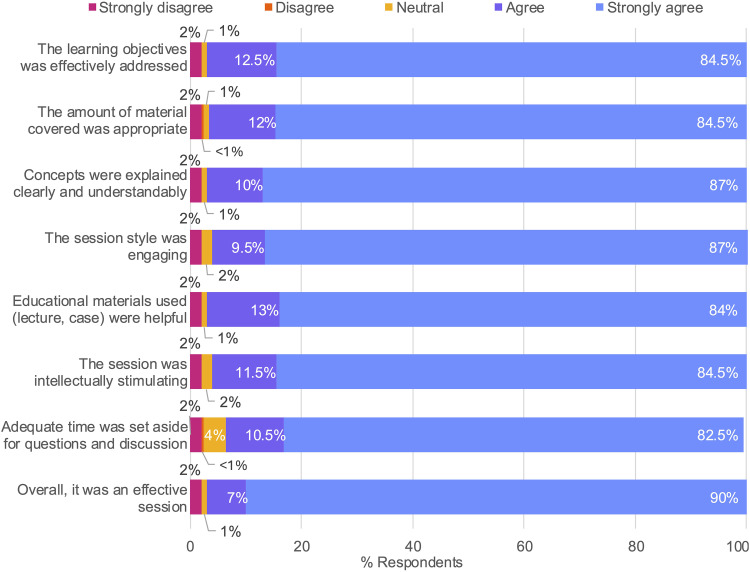
Graphical representation of Likert scale session evaluation across all sessions.

Postsession feedback surveys were overall positive (Figure 4). Most (>83%) participants strongly agreed that learning objectives were effectively addressed, the educational materials used (lecture, CBL case, procedural workshop) were helpful, the amount of material covered was appropriate, the concepts were explained clearly and understandably, and there was adequate time for questions and discussion.

Across all sessions, we received 73 written feedback responses. Thematic analysis of open-ended responses revealed 4 overarching themes: (1) experiential learning and clinical/technical skill confidence, (2) curriculum and content relevance, (3) engagement through multimodal and interactive teaching, and (4) learning environment and mentorship, as detailed in Table 3.

**Table 3.** Themes and Select Quotes of Student Feedback.

Theme	Select Quotes
(1) Experiential learning and clinical/technical skill confidence	“The hands-on session was very engaging and absolutely helped to maintain the information in my mind!” “More time for hands-on skills” “Loved it! Great to have the opportunity to have real experience with the tools and techniques taught.” “Love the suturing lesson!”; “Great job!! The biopsy activity was a hit.” “The splinting activity was my favorite.” “The (incision and drainage) activity was awesome!”
(2) Curriculum and content relevance	“Lecture was amazing! It broke a lot of my misconceptions about plastic surgery, and I learned how it can truly be life-changing.” “(The session) paired very well with the content we are learning and piqued interest in plastics.” “Amazing session that should be open to all students without having to sign up for something external to the school schedule.” “Awesome session. Thanks for teaching the reconstructive ladder!” “Every session is insightful and engaging. I always walk away with a learning token that helps me with shadowing!” “Would love more surgical content in the (*Medical Foundations [ie pre-clerkship*]) curriculum.”
(3) Engagement through multimodal and interactive teaching	“The combination of lecture, case-based learning (CBL), and hands-on skills is extremely engaging!” “It was really great! Loved the mix of interactive and didactic teaching in the session.” “(The slides) were very clear and helpful.”
(4) Learning environment and mentorship	“Great session … casual but lots of chances to ask questions and get feedback.” “Thanks for facilitating a welcoming atmosphere and providing opportunities to ask questions and learn!” “It was really interesting, loved that it came from the perspective of residents, thank you so much for teaching us how to do stitches and being so kind.” “More time for Q&A with residents and surgeons.” “Wonderful session and leaders!!”

#### Theme 1: Experiential Learning and Clinical/Technical Skill Confidence

Participants consistently highlighted the hands-on workshop as the most valuable aspect of the sessions. Workshops were described as engaging, memorable, and effective for consolidating learning. One participant noted, “The hands-on session was very engaging and absolutely helped to maintain the information in my mind!” Several participants emphasized the value of repetition and variety, requesting “More time for hands-on skills.”

#### Theme 2: Curriculum and Content Relevance

Students noted that sessions complemented medical school coursework and clarified misconceptions about PRS. One respondent reported that “Lecture was amazing! It broke a lot of my misconceptions about plastic surgery, and I learned how it can truly be life-changing.” Another suggested integration into the formal pre-clerkship curriculum: “Amazing session that should be open to all students without having to sign up for something external to the school schedule,” and “Would love more surgical content in the (*Medical Foundations [ie pre-clerkship*]) curriculum.”

#### Theme 3: Engagement Through Multimodal and Interactive Teaching

Students consistently highlighted the multimodal format, combining didactic lecture, CBL, and hands-on skills, as highly engaging and effective. One respondent shared that “The combination of lecture, CBL, and hands-on skills is extremely engaging!” while another noted, “It was really great! Loved the mix of interactive and didactic teaching in the session.” Learners also emphasized the quality of the visual materials, commenting that “(The slides) were very clear and helpful.”

#### Theme 4: Learning Environment and Mentorship

Overall, the sessions were described as welcoming, collegial, and motivating. One response reported that the sessions were “casual but lots of chances to ask questions and get feedback.” Many students valued opportunities to interact with residents and staff: “Loved that (the workshop) was led by residents, thank you so much for teaching us how to do sutures,” and requesting “more time for Q&A with residents and surgeons.”

## Discussion

This study describes the implementation of a multimodal, in-person PRS-CBL curriculum and presents 2 years of participation, evaluation, and short-term knowledge outcomes. The sessions drew consistent attendance across both years, and students frequently highlighted the hands-on and case-based components as valuable aspects of their learning experience. The curriculum's structure, which combines didactic teaching, cases, and procedural skills, was designed to parallel the existing pre-clerkship curriculum at our institution, and the observed engagement suggests that this format is well suited for the learners’ level of training.

Several strategies and initiatives have been proposed to increase access and early exposure to PRS among medical students worldwide. Examples include the PRS Virtual Curriculum, a 3-month virtual curriculum designed for third-year medical students.^22^ Within Canada, several PRS-focused educational initiatives have also been described. The University of British Columbia offers a series of educational YouTube videos specifically designed for medical students.^41^ The University of Calgary provides primer sessions covering topics such as hand fractures, wound reconstruction, and cranial fractures.^42^ Finally, the PRS Community (PRSC), a national student-led organization, provides educational content on social media and delivers individual lectures hosted by staff plastic surgeons throughout the academic year.^43^

Within this broader landscape, PRS-CBL contributes a structured, pre-clerkship, in-person, multimodal PRS curriculum intentionally aligned with the local undergraduate medical curriculum. First, the sessions were designed to parallel and complement the formal pre-clerkship medical curriculum, to ensure the content was tailored to first- and second-year medical students’ level of training. At our institution, CBL is already well-incorporated into pre-clerkship education, making this format a natural extension of curricular structure. Second, the sessions were delivered entirely in-person, facilitating active engagement, hands-on learning, and meaningful interactions with peers, residents, and faculty. The in-person, small-group, and interactive sessions fostered early, low-barrier mentorship opportunities through one-on-one dialogue, informal career guidance, and the development of longitudinal professional connections (Theme 4, Table 3). Early exposure to mentorship has been shown to play a significant role in medical students’ specialty exploration and career decision-making, which is an advantage that is difficult to achieve through virtual or asynchronous educational platforms.^14,16,44,45^ Finally, the PRS-CBL curriculum was delivered in a multimodal format, combining didactic teaching, interactive case discussions, and hands-on workshops in every session, highlighting both the academic and applied portions of medical education. From a pedagogical perspective, this approach aligns with Bloom's Taxonomy framework, supporting a progression through increasingly complex levels of cognitive engagement.^46^ The didactic components of each session address foundational knowledge and comprehension, while case discussions promote application and analysis of clinical reasoning. The hands-on technical components engage students in the higher-order psychomotor domains of Bloom's framework by requiring integration of anatomical knowledge, clinical judgment, and procedural skills with real-time feedback from peers and facilitators. Educational theory suggests that engaging multiple cognitive domains, particularly higher-order processes such as applying, analyzing, and creating, enhances long-term retention and transferability of knowledge.^47^

Early exposure to PRS may be particularly valuable during pre-clerkship training, when many medical students remain undifferentiated in their career interests and are gradually narrowing their specialty preferences.^2,15,48–50^ In the present study, participants reported broad interest across both medical and surgical specialties, likely reflecting this stage of training. Exposure to structured PRS educational initiatives and informal mentorship during this period may help contextualize the specialty and reduce perceived barriers to engagement for early trainees. Importantly, the widespread engagement with the PRS-CBL initiative also suggests that high-yield PRS knowledge is relevant not only to students considering a career in PRS, but also to those pursuing other specialties. This further supports broader integration of PRS into preclinical curricula. A comparable initiative at the University of Glasgow Wolfson Medical School reached a similar conclusion and based on student feedback, PRS teaching was subsequently integrated into the core medical curriculum.^51^

Over the 2-year implementation period, considerable growth in the number of registrants and attendees was observed. We hypothesize that several factors contributed to this pattern. In the first year of implementation, relatively lower multisession participation may have reflected the optional nature of the curriculum, scheduling and examination conflicts within the pre-clerkship academic calendar, competing student commitments, and the early stage of program development. In the second year, increased attendance and longitudinal retention coincided with several refinements to the curriculum, including closer alignment of session timing with the pre-clerkship academic schedule, expanded communication and outreach efforts, and the inclusion of hands-on components at each session. The addition of a certificate of completion, signed by faculty in the Division of PRS at our institution, may also have encouraged some students to attend multiple sessions, although its specific impact cannot be quantified. We further hypothesize that positive peer-to-peer dissemination of experiences also contributed to this increase, whereby participants informally shared favorable perceptions of the curriculum's relevance, accessibility, and educational value with subsequent cohorts.

With respect to knowledge outcomes, the absence of a statistically significant improvement in knowledge scores following session 4 may reflect session-specific factors, including content complexity, assessment design, lower response rates, or variability in participant engagement. This may also be explained by the smaller sample size and greater variability in responses. This finding identifies an area for ongoing curriculum refinement, and future evaluation should explore more focused evaluation of session design and learner experience to better understand how the session and its assessment can be optimized through a learner-centred approach. Overall, the pattern of participation suggests ongoing interest in structured PRS teaching early in medical training. See Table 4 for an outline of iterative refinements aimed to improve multisession registration and retention across academic years.

**Table 4.** Iterative Curriculum Promotional, Organizational, and Delivery Refinements to Support Multisession Plastic and Reconstructive Surgery Case-Based Learning (PRS-CBL) Participation and Longitudinal Retention.

Changes From Year 1 to Year 2
➢ Closer alignment of session timing with the pre-clerkship academic schedule, reducing scheduling and exam conflicts ➢ Expanded communication and outreach efforts ➢ Inclusion of hands-on components at each session ➢ Introduction of a certificate of completion for students attending at least 4 of 5 sessions and completing the required postsession surveys
Changes Implemented in Year 3
➢ Structured social media campaign and posting schedule ➢ Invitation-to-register emails to students who had previously registered for a session that academic year ➢ Broader session promotion through the institution-affiliated PRS Instagram page ➢ Use of QR codes to direct students to feedback forms ➢ Streamlining of postsession survey completion as a prerequisite for attendance to count toward certificate eligibility, including a link to the attendance form displayed after survey submission ➢ Cross-checking session dates with divisional activities to reduce scheduling conflicts

The increased attendance and longitudinal retention of students in our second year can be attributed to both iterative modifications to the curriculum and positive peer-to-peer dissemination of experiences, whereby participants informally shared favorable perceptions of the curriculum's relevance, accessibility, and educational value with students of subsequent cohorts. This aligns with established models of social learning and diffusion of educational innovations in medical training.^52^ To further extend its reach, an Instagram account was also developed to engage more medical students and to share educational content created through the curriculum. Additionally, integrating PRS-CBL into the formal pre-clerkship curriculum would ensure consistent exposure to PRS concepts for all medical students, thereby reducing reliance on voluntary participation. The model may be adopted by other institutions seeking to increase awareness of PRS among pre-clerkship learners and to promote more equitable access to early surgical education opportunities. Its broader applicability would require evaluation within different curricular structures, but the core multimodal format offers a feasible framework for institutions aiming to enhance introductory PRS teaching.

Although the PRS-CBL initiative has demonstrated growing student engagement and measurably improved educational impact, several challenges emerged throughout its development and growth. During the initial year of implementation, student participation was limited, with relatively low registration per session. To address this, session structure and outreach strategies were refined, including the addition of hands-on components and alignment with the pre-clerkship academic calendar and exam schedules. These adaptations led to a marked increase in engagement in the second year, with sessions consistently reaching maximum capacity. Paradoxically, the initiative's implementation introduced new considerations (and our PRS-CBL curriculum fell victim to its early success), such as large participant numbers limiting individualized learner interaction and instruction, making small-group facilitation more challenging. Furthermore, despite strong attendance and alignment with pre-clerkship curricular objectives, efforts to formally integrate the PRS-CBL model into the core pre-clerkship medical curriculum have encountered limited institutional uptake.

This study has several limitations. As a single-institution initiative, these findings may not be generalizable to all medical schools, particularly those with differing curricula or student demographics, such as institutions without home PRS programs. Participation was voluntary, which may have introduced selection bias, as students already interested in PRS or surgery may have been over-represented. Although short-term knowledge gains were measured, the study did not assess long-term retention or application of knowledge in clinical contexts. Additionally, the outcome measures used in this study, including the knowledge quizzes and session evaluation surveys, were locally developed and not formally validated, which may limit the precision and interpretability of the findings. Finally, as noted in the Methods, presession and postsession surveys could not be matched, precluding within-participant comparisons and potentially reducing statistical power.

## Conclusion

This study highlights the educational value of a structured, in-person, multimodal PRS teaching curriculum for pre-clerkship medical students. By enhancing knowledge, promoting interest in PRS, and offering networking opportunities, the program addresses a gap in early PRS education and exposure. The favorable evaluations and short-term knowledge gains suggest that similar multimodal teaching models could be explored in other underrepresented specialties to support early exposure within pre-clerkship medical education. This would contribute to more informed decision-making processes in specialty selection and holistic preparation for medical practice. Future work will aim to advocate for formal integration of a modified PRS-CBL curriculum into the first- and second-year undergraduate medical curriculum to provide standardized pre-clerkship exposure to foundational PRS principles.

## Supplemental Material

sj-docx-1-psg-10.1177_22925503261470691 - Supplemental material for Development and Delivery of a Plastic and Reconstructive Surgery Case-Based Learning Curriculum for Pre-Clerkship Medical Students Supplemental material, sj-docx-1-psg-10.1177_22925503261470691 for Development and Delivery of a Plastic and Reconstructive Surgery Case-Based Learning Curriculum for Pre-Clerkship Medical Students by Dave Gwun, Victoria Tucci, Alexandra D'Souza, Thomas Milazzo, Sophocles Voineskos, David Wallace, Kyle R. Wanzel and Shaishav Datta in Plastic Surgery

sj-docx-2-psg-10.1177_22925503261470691 - Supplemental material for Development and Delivery of a Plastic and Reconstructive Surgery Case-Based Learning Curriculum for Pre-Clerkship Medical Students Supplemental material, sj-docx-2-psg-10.1177_22925503261470691 for Development and Delivery of a Plastic and Reconstructive Surgery Case-Based Learning Curriculum for Pre-Clerkship Medical Students by Dave Gwun, Victoria Tucci, Alexandra D'Souza, Thomas Milazzo, Sophocles Voineskos, David Wallace, Kyle R. Wanzel and Shaishav Datta in Plastic Surgery

sj-docx-3-psg-10.1177_22925503261470691 - Supplemental material for Development and Delivery of a Plastic and Reconstructive Surgery Case-Based Learning Curriculum for Pre-Clerkship Medical Students Supplemental material, sj-docx-3-psg-10.1177_22925503261470691 for Development and Delivery of a Plastic and Reconstructive Surgery Case-Based Learning Curriculum for Pre-Clerkship Medical Students by Dave Gwun, Victoria Tucci, Alexandra D'Souza, Thomas Milazzo, Sophocles Voineskos, David Wallace, Kyle R. Wanzel and Shaishav Datta in Plastic Surgery

**Figure:** Video 1.
