# Supplementary material for: Development and Delivery of a Plastic and Reconstructive Surgery Case-Based Learning Curriculum for Pre-Clerkship Medical Students
Source: Plast Surg (Oakv). 2026 Jul 30:22925503261470691. Online ahead of print. doi: 10.1177/22925503261470691 (PMC13458317; doi:10.1177/22925503261470691)
Supplement: Supplementary file 1 [file sj-docx-1-psg-10.1177_22925503261470691.docx]

| **Supplemental Material S1:** The Guideline for Reporting Evidence-based practice Educational interventions and Teaching (GREET) checklist* | page |
| --- | --- |
| BRIEF NAME |  |
| 1. INTERVENTION: Provide a brief description of the educational intervention for all groups involved [e.g. control and comparator(s)]. | 8-11 |
| WHY - this educational process |  |
| 2. THEORY: Describe the educational theory (ies), concept or approach used in the intervention. | 6-7, 16 |
| 3. LEARNING OBJECTIVES: Describe the learning objectives for all groups involved in the educational intervention. | Table 1 |
| 4. EBP CONTENT: List the foundation steps of EBP (ask, acquire, appraise, apply, assess) included in the educational intervention. | NA |
| WHAT |  |
| 5. MATERIALS: Describe the specific educational materials used in the educational intervention. Include materials provided to the learners and those used in the training of educational intervention providers. | 8-10 |
| 6. EDUCATIONAL STRATEGIES: Describe the teaching / learning strategies (e.g. tutorials, lectures, online modules) used in the educational intervention. | 8-10 |
| 7. INCENTIVES: Describe any incentives or reimbursements provided to the learners. | 10 |
| WHO PROVIDED |  |
| 8. INSTRUCTORS: For each instructor(s) involved in the educational intervention describe their professional discipline, teaching experience / expertise. Include any specific training related to the educational intervention provided for the instructor(s). | 9 |
| HOW |  |
| 9. DELIVERY: Describe the modes of delivery (e.g. face-to-face, internet or independent study package) of the educational intervention. Include whether the intervention was provided individually or in a group and the ratio of learners to instructors. | 8-10 |
| WHERE |  |
| 10. ENVIRONMENT: Describe the relevant physical learning spaces (e.g. conference, university lecture theatre, hospital ward, community) where the teaching / learning occurred. | 9 |
| WHEN and HOW MUCH |  |
| 11. SCHEDULE: Describe the scheduling of the educational intervention including the number of sessions, their frequency, timing and duration. | 8-10, Table 1, Figure 1 |
| 12. Describe the amount of time learners spent in face to face contact with instructors and any designated time spent in self-directed learning activities. | 8-10 |
| PLANNED CHANGES |  |
| 13. Did the educational intervention require specific adaptation for the learners? If yes, please describe the adaptations made for the learner(s) or group(s). | No |
| UNPLANNED CHANGES |  |
| 14. Was the educational intervention modified **during** the course of the study? If yes, describe the changes (what, why, when, and how). | Yes, 18 |
| HOW WELL |  |
| 15. ATTENDANCE: Describe the learner attendance, including how this was assessed and by whom. Describe any strategies that were used to facilitate attendance. | 10,  Figure 2 |
| 16. Describe any processes used to determine whether the materials (item 5) and the educational strategies (item 6) used in the educational intervention were delivered as originally planned**.** | NA |
| 17. Describe the extent to which the number of sessions, their frequency, timing and duration for the educational intervention was delivered as scheduled (item 11). | 8-10 |

***Based on the TIDieR guidance.**
