# Supplementary material for: Development and Delivery of a Plastic and Reconstructive Surgery Case-Based Learning Curriculum for Pre-Clerkship Medical Students
Source: Plast Surg (Oakv). 2026 Jul 30:22925503261470691. Online ahead of print. doi: 10.1177/22925503261470691 (PMC13458317; doi:10.1177/22925503261470691)
Supplement: Supplementary file 2 [file sj-docx-2-psg-10.1177_22925503261470691.docx]

**Supplemental Material S2:** Sample pre-session registration survey.

# Plastic and Reconstructive Surgery Case-Based Learning Session 1: Introduction to Plastic Surgery & Suturing Workshop (2025-2026)

## Event Details:

Date: ***

Time: ***

Format: In-person

Location: ***

(Room and floor details will be shared following registration.)

## Session Overview

This case-based learning session is part of a longitudinal series designed to increase pre-clerkship exposure to high-yield Plastic and Reconstructive Surgery (PRS) topics. Developed as an informal but complementary curriculum, these sessions aim to strengthen foundational knowledge in PRS through interactive case discussions and hands-on skill development.

This session will be co-facilitated by *** PRS residents:

• *** (PGY3)
• *** (PGY2)
• *** (PGY1)

The first portion of the evening will introduce core principles in Plastic Surgery, followed by a suturing workshop.

Eligibility: Open to first- and second-year medical students only.
Registration deadline: ***.

For questions, contact: ***

Participation is voluntary and responses are anonymous. By submitting this form, you consent to the use of anonymized responses for analysis and research dissemination.

## Registration Information

Name: ________________________________

Email: ________________________________

Year of Medical School Graduation: □ 2028 □ 2029 □ MD/PhD □ Other: __________

Specialty/Specialties of Interest: ___________________________

Questions or Concerns: ___________________________

Do you consent to photo use on PRS-CBL social media? □ Yes □ No

## Demographic Survey

We are collecting limited demographic information (age, sex, and ethnicity/race) to better understand the diversity of participants and to ensure that our findings are representative and meaningful across different groups. This information will help us identify potential differences in experiences or outcomes that may otherwise be overlooked. All demographic data will be anonymized, kept strictly confidential, stored securely, and reported only in aggregate form so that no individual participant can be identified.

Age: __________

Gender: □ Female □ Male □ Transgender □ Non-binary/Gender-diverse □ Prefer not to disclose □ Other: __________

Ethnicity (select all that apply):

□ African/Black (including African-American, African-Canadian, Caribbean)

□ East Asian (e.g., Chinese, Taiwanese, Japanese, Korean, etc.)

□ European/White

□ Indo-Caribbean, Indo-African, Indo-Fijian, West-Indian

□ Latin, South or Central American

□ Polynesian (e.g., Samoan, Tongan, Cook Island Maori, Tahitian, Hawaiian, Maori)

□ South Asian (e.g., Indian, Pakistani, Bangladeshi, Sri Lankan, Nepali, Tamil, etc.)

□ Southeast Asian (e.g., Vietnamese, Thai, Filipino/a, Malaysian, etc.)

□ West Asian (e.g., Iranian, Lebanese, Turkish, Palestinian, etc.)

□ Indigenous within Canada (First Nation, Métis, Inuit)

□ Prefer not to answer

□ Other: __________

## Knowledge Questions

Please answer the following formative knowledge questions. Responses are FORMATIVE, no prior knowledge or understanding is required, so please do not use external materials to answer the following questions.

1. Who is considered the father of plastic surgery and defined it as “the art devoted to restore what nature has given and chance has taken away”?

□ Gaspare Tagliacozzi □ Harold Gillis □ Leith Douglas □ Ross Tilley

2. Which of the following is true with respect to skin wound healing?

□ Time of injury to day 4 represents the inflammatory phase of wound healing
□ The proliferative phase involves hemostasis, vascular permeability, and macrophage infiltration
□ The proliferative phase lasts from approximately day 3 to week 3
□ Wound strength decreases during the remodeling phase

3. Which of the following is not part of the reconstructive ladder?

□ Delayed Primary Closure □ Secondary Intention □ Delayed Secondary Closure □ Dermal Substitutes
