# Supplementary material for: Development and Delivery of a Plastic and Reconstructive Surgery Case-Based Learning Curriculum for Pre-Clerkship Medical Students
Source: Plast Surg (Oakv). 2026 Jul 30:22925503261470691. Online ahead of print. doi: 10.1177/22925503261470691 (PMC13458317; doi:10.1177/22925503261470691)
Supplement: Supplementary file 3 [file sj-docx-3-psg-10.1177_22925503261470691.docx]

# Supplemental Material S3: Sample post-session feedback survey.

# Plastic and Reconstructive Surgery Case-Based Learning Feedback Survey: Introduction to Plastic Surgery Session (2025–2026)

Thank you for attending the PRS-CBL Introduction to Plastic Surgery Session, led by ***.

Please take a few minutes to complete this anonymous feedback form. Your responses help us improve future sessions.

## Medical School Graduation Year

□ 2028 □ 2029 □ Other: ___________________________

**Please rate your agreement with the following statements:**

The learning objectives were effectively addressed.
□ Strongly Disagree □ Disagree □ Neutral □ Agree □ Strongly Agree

The amount of material covered was appropriate.
□ Strongly Disagree □ Disagree □ Neutral □ Agree □ Strongly Agree

Concepts were explained clearly and understandably.
□ Strongly Disagree □ Disagree □ Neutral □ Agree □ Strongly Agree

The session style was engaging.
□ Strongly Disagree □ Disagree □ Neutral □ Agree □ Strongly Agree

The educational materials used (lecture, case, hands-on workshop) were helpful.
□ Strongly Disagree □ Disagree □ Neutral □ Agree □ Strongly Agree

The session was intellectually stimulating.
□ Strongly Disagree □ Disagree □ Neutral □ Agree □ Strongly Agree

Adequate time was set aside for questions and discussion.
□ Strongly Disagree □ Disagree □ Neutral □ Agree □ Strongly Agree

Overall, it was an effective session.
□ Strongly Disagree □ Disagree □ Neutral □ Agree □ Strongly Agree

Any feedback or suggestions for improvement:

_________________________________________________________

## Knowledge Questions (Formative)

Please answer the following formative questions. These are for learning purposes only.

1. Who is considered the father of plastic surgery and defined it as “the art devoted to restore what nature has given and chance has taken away”?

□ Gaspare Tagliacozzi □ Harold Gillis □ Leith Douglas □ Ross Tilley

2. Which of the following is true with respect to skin wound healing?

□ Time of injury to day 4 is the time period for the inflammatory phase of wound healing
□ The proliferative phase of wound healing involves hemostasis, increased vascular permeability, and macrophage infiltration
□ The proliferative phase of wound healing lasts from ~day 3 to ~week 3
□ Wound strength decreases in the remodeling phase of wound healing

3. Which of the following is NOT a part of the reconstructive ladder?

□ Delayed Primary Closure □ Secondary Intention □ Delayed Secondary Closure □ Dermal Substitutes

Thank you for completing this survey! Your feedback is greatly appreciated.
